# RNA-seq based transcriptome analysis of ethanol extract of saffron protective effect against corticosterone-induced PC12 cell injury

**DOI:** 10.1186/s12906-022-03516-1

**Published:** 2022-01-31

**Authors:** Xi Chen, Ting Yang, Congen Zhang, Zhijie Ma

**Affiliations:** 1 Department of Pharmacy, Beijing Friendship Hospital, Capital Medical University, Beijing, 100050 China; 2 Department of Pharmacology, Capital Medical University School of Basic Medical Sciences, Beijing, 100069 China

**Keywords:** Ethanol extract of crocus, Depression, RNA-seq, MAPK signaling pathway, qPCR

## Abstract

**Background:**

At present, oral antidepressants are commonly used in the clinical treatment of depression. However, the current drug treatment may lead to more serious adverse reactions. Therefore, we focus on Chinese traditional medicine, trying to find an effective and safe alternative or complementary medicine. *Crocus sativus* (saffron) is a traditional Chinese herbal medicine, which is typically used in the clinic to regulate anxiety, insomnia, amnesia, and other mental disorder. The study aimed to explore the neuroprotective effect of ethanol extract of saffron (EES) on corticosterone (CORT)- induced injury in PC12 cells and further explored its potential mechanism.

**Methods:**

The authenticity of saffron and the active components of EES were identified by a water test and ultra-performance liquid chromatography-time of flight mass spectrometry system. The screening of cytotoxicity for PC12 cells was incubated with EES in different concentrations for 24 h, and the protective efficacy of EES on CORT (500 μM) -induced PC12 cell injury, cell viability was assessed by Cell Counting Kit-8 (CCK-8) assay. The differentially expressed genes (DEGs) of EES-protected PC12 cells were analyzed using the RNA-seq method, and the results were analyzed for GO and KEGG enrichment. The results of RNA-seq were verified by qPCR analysis.

**Results:**

The saffron was initially identified as authentic in the water test and 10 compounds were identified by Ultra Performance Liquid Chromatography (UPLC)- Mass Spectrometry (MS). The results of CCK-8 demonstrated that EES at concentrations above 640 μg/mL exerted a certain cytotoxic effect, and PC12 cells pretreated with EES (20, 40, and 80 μg/mL) significantly reversed the 500 μM CORT-induced cell death. RNA-seq analysis showed that EES regulated 246 differential genes, which were mainly enriched in the MAPK signaling pathway. Dusp5, Dusp6, Gadd45b, Gadd45G, and Pdgfc were further validated by qPCR. Experimental data showed that the results of qPCR were consistent with RNA-seq.

**Conclusions:**

These findings provide an innovative understanding of the molecular mechanism of the protective effect of EES on PC12 cells at the molecular transcription level, and Dusp5, Dusp6, Gadd45b, Gadd45g, and Pdgfc may be potential novel targets for antidepressant treatment.

## Background

Depression is a severe mental disorder that afflicts many people. It is characterized by low spirit, loss of interest in life, and unexplained pain. According to the data of the World Health Organization, there are over 300 million people worldwide suffer from depression [1]. Patients with depression often suffer from sadness, fatigue, crying, pessimism, changes in sleep patterns, and lack of pleasant sensation, making depression the most important cause of disability due to the disease [2].

At present, oral antidepressants are commonly used in the clinical treatment of depression. Specifically, selective serotonin reuptake inhibitors (SSRIs) are the most widely used antidepressants in the clinic and are the first-line treatment for depression. However, current antidepressants can cause serious adverse reactions in patients. The most troubling adverse reactions seem to be gastrointestinal problems, sleep disorders, and sexual dysfunction [3]. At the same time, withdrawal symptoms after drug discontinuation can lead to even worse conditions in patients [4].

*Crocus sativus L.* is a perennial herb commonly known as saffron. Saffron has been used as a spice and medicinal material for many years and as a drug for 3600 years [5]. Notably, saffron and its main components and secondary metabolites have been proved to have antioxidant [6], neuroprotective [7], and antidepressant effects [8, 9], which have been preliminarily verified in clinical practice. The antidepressant mechanism of saffron may be derived from its anti-oxidative stress effect on cells. A meta-analysis of seven randomized controlled trials showed that the ingestion of saffron significantly reduced serum malondialdehyde and increased total antioxidant capacity, highlighting the positive effect that saffron appeared to have on Oxidative stress [10].

The current basic research shows that saffron has extremely low toxicity, which provides a better guarantee for the safety of long-term treatment of depression patients with saffron [11].

Currently, differentiated PC12 cells have been widely used as an in vitro model in neurobiological or neuromuscular mechanisms underlying the antidepressant effect of drugs. Correspondingly, we selected corticosterone to induce injury to PC12 cells. It is the major glucocorticoid in rodents [12]. Excessive corticosterone exposure could activate hippocampal negative feedback regulation instructions and inhibit the activity of the hypothalamic-pituitary-adrenal (HPA) axis [13]. In particular, multiple studies have reported that continuous exposure to high concentrations of CORT can lead to pathological hippocampal neuronal damage and induce depressive-like behavior due to HPA feedback dysfunction [14]. Therefore, the coculture of PC12 with CORT is well-established in vitro model for studying depression and nerve injury.

RNA-seq is a new technique in transcriptome research. The technology combines an experimental method of transcriptome sequencing and a database information analysis method of a digital gene expression spectrum. RNA-seq technology has the advantages of high sensitivity, high throughput, high precision, and a wide detection range [15]. It can provide a basic perspective on the understanding of genomic organization and regulation, which in turn can provide valuable information on the intracellular state and the impact of changes in the expression of mutated genes on the study of complex diseases. Elucidation of changes in gene expression is fundamental to understanding the cellular response to internal genetic lesions, external stimuli, and changing environmental conditions. Consequently, RNA expression profiles are a unique source of biomarkers for the prediction and classification of human diseases [16]. Therefore, RNA-seq contributes to revealing comprehensive and specific changes in the transcriptome and better studying the protective mechanism of the saffron extract on corticosterone-induced PC12 cell injury.

The core of our study was to further explore whether saffron has antidepressant activity as well as the potential for the treatment of depression, and then to search for its possible molecular mechanism. This may become a better solution to the many adverse drug events of current clinical antidepressants. Furthermore, it is expected that the vast numbers of healthy individuals, patients, and clinicians patients will have safer, more economical, and effective coping strategies when faced with, depressed mood, pessimism, and depression.

## Methods

### Identification of saffron by water test

After saffron was immersed in water at room temperature, there would appear obvious physicochemical phenomena, which could be used to identify the authenticity of the variety. The dried stigma of saffron (0.5 g), identified by Professor Zhao Kuijun from Beijing Friendship Hospital, Capital Medical University, was weighed and floated on the water, and the color change of water and the morphological change of medicinal material was recorded at different time points.

### Preparation of sample solutions

Ethanol extract of saffron (EES) was extracted by referring to the method in the criterion of the Pharmacopoeia of the People’s Republic of China, 2015. To be specific, the saffron (1 g) was accurately weighed, pulverized, added with 10 mL of 75% ethanol, then, ultrasonically extracted in an ice bath. After 2 h, the EES Freeze-dried powder was collected by vacuum freeze dryer. All dried samples were powdered to a homogeneous size and sieved through a No. 65 mesh. In each case, 0.1 g of powder was ultrasonic-extracted for 20 min with 50 mL of methanol: water (1:1). The supernatant solution was filtered through a 0.2 mm poly tetra fluoroethylene (PTFE) membrane filter before being injected into the UPLC system.

### Content determination and component identification of ethanol extract of saffron

Chromatographic experiments were performed as described previously. Briefly, Acquity UPLC system (Waters, Milford, MA) connected to Xevo G2 Q-ToF MS equipped with an electrospray ionization source (Waters, Milford, MA, USA) was performed on an ACQUITY UPLC T3 column (2.1× 100 mm,1.7 μm) by gradient elution. The column temperature was maintained at 45 °C and the flow was 0.4 mL/min. The mobile phase consisted of solution A (water with 0.1% formic acid) and solution B (acetonitrile with 0.1% formic acid). Elution started at 5% B which was held constant for 1 min, then increased linearly to 100% from 1 to 16 min, and the injection volume was 5 μL.

The Electrospray ionization Quadrupole Time-of-Flight Mass Spectrometer (ESI-Q-TOF-MS) detection was operated using the positive (ESI+) ion mode in the mass range of 50–2000 Da. The optimized mass spectrometer parameters were set as follows: capillary voltage of 2.5 kV, sample cone voltage of 21 V, source and desolvation temperatures were 130 °C and 350 °C respectively. Cone gas flow of 50 L/h, desolvation gas flow of 800 L/h, nitrogen, and argon was used as the cone and collision gases. Data were switched between the low energy (4 V) and elevated energy (10–55 V) acquisition modes every 0.2 s.

### Cell culture

PC12 cells were obtained from the Shanghai Institute of Biochemistry and Cell Biology (Shanghai, China). They were cultured in RPMI1640 medium (Gibco, Grand Island, NY, USA) supplemented with 5% fetal bovine serum (HyClone, Logan, UT, USA), 100 mg/ml streptomycin, and 100 U/ml penicillin at 37 °C in a humidified atmosphere with 5% CO_2_.

### In vitro cytotoxicity of EES

For the cell experiments, EES was dissolved in water, prepared into a 2 mg/mL stock solution (calculated based on the concentration of raw medicinal materials). The screening of cytotoxicity for PC12 cells was assessed by Cell Counting Kit-8 (CCK-8) assay kit (Dojindo, Kumamoto, Japan) as previously described. Briefly, the PC12 cells were placed into a 96-well cell culture cluster at 4 × 10^3^ per well. Then, the cells were treated with EES in different concentrations. After 24 h, 10 μL CCK-8 was added to each sample respectively, and the cells were incubated at 37 °C for 1 h. With mixing gently to ensure homogeneous distribution of color, the absorbances of solution were measured at 450 nm performing a microplate reader (Molecular Devices, USA). The cell was calculated using the equation: cell viability (100%) = (OD_treatment_/ OD_control_) × 100%.

### Protective efficacy of EES on corticosterone induced PC12 cell injury

The PC12 cell culture method was the same as above. After attachment, respectively, cells were incubated with different concentrations (10, 20, 40, 80, 160, 320, and 640 μg/mL) of EES for an additional 24 h. To validate the experiments, fluoxetine (10 μM, Sigma-Aldrich, St. Louis, MO, USA), a classical antidepressant, was used as a positive control. Then, cells were coincubated with 500 μM of corticosterone (Sigma-Aldrich, St. Louis, MO, USA) for 24 h. Finally, the calculation method of cell viability rate is the same as above. Optimal EES concentrations for use in the following experiments were determined based on preliminary results.

### Total RNA isolation, library construction, and mRNA sequencing

After corresponding treatment, PC12 cells were lysed in TRIzol reagent (Invitrogen, USA) and total RNA was extracted. A total amount of 2 μg RNA per sample was used as input material for the RNA sample preparations. Sequencing libraries were generated using NEBNext UltraTM RNA Library Prep Kit for Illumina (NEB, USA) following the manufacturer’s recommendations and index codes were added to attribute sequences to each sample. Briefly, mRNA was purified from total RNA using poly-T oligo-attached magnetic beads. Fragmentation buffer was added for interrupting mRNA to short fragments. First-strand cDNA was synthesized with random hexamers using the fragments as templates. Then, Buffer, dNTPs, RNase H, and DNA polymerase I was added to synthesize the second-strand cDNA. The double-stranded cDNA was then purified, end-repaired, and A-tailed was added for adaptor ligation. DNA fragments of 250–300 bp in length were selected preferentially by purifying the library fragments with the AMPure XP system (Beckman Coulter, Beverly, USA). Finally, PCR amplification was performed to enrich the cDNA libraries, whose quality was assessed on the Agilent Bioanalyzer 2100 system. After cluster generation, the library preparations were sequenced on an Illumina Hiseq 4000 platform and paired-end 150 bp reads were generated.

1. Data analysis of DEGs

The DEGs were analyzed using the DESeq R package (version 1.10.1), which has been a routine statistical approach to determine the digital differential expressions of genes using a model assuming the negative binomial distribution of the data. To control the false positive rate, Benjamini and Hochberg’s approach was used for adjusting the *P* values, and a *P* value less than 0.05 was considered to indicate a significant difference.

2. Functional enrichment analysis

The DEGs were subjected to enrichment analysis of Gene Ontology (GO) and Kyoto Encyclopedias of Genes and Genomes (KEGG). GO functional enrichment and KEGG pathway enrichment were performed by Metascape (https:// metascape.org) [17].

3. Confirmation of RNA-seq results with qPCR analysis

PC12 cells were lysed in TRIzol reagent as above. For PCR analysis, cDNA was Synthesized via FastKing RT Kit (With gDNase) (TIANGEN BIOTECH, Cat: KR116). Amplification and quantitation of PCR were performed using a real-time PCR system with SuperReal PreMix Plus (SYBR Green) (TIANGEN BIOTECH, Cat: FP205). mRNA expression levels were normalized to GAPDH mRNA levels. Fold expression determination, gene-to-GAPDH ratios were determined by using the 2^−ΔΔCt^ method. The thermal cycles were performed with an initial denaturation at 95 °C for 10 min, followed by 40 cycles of 95 °C for 15 s and 60 °C for 1 min.

### Statistical analysis

As detailed above, statistical analysis and graphs were performed with GraphPad Prism 8.3 software (GraphPad Software, La Jolla, CA, USA). Results were demonstrated as mean ± standard deviation (SD). Statistical significance was determined by one-way analysis of variance followed by Tukey’s secondary test for significance. *P* < 0.05 was considered to be statistically significant.

## Result

### Identification and observation of saffron in water test

**Fig. 1:**
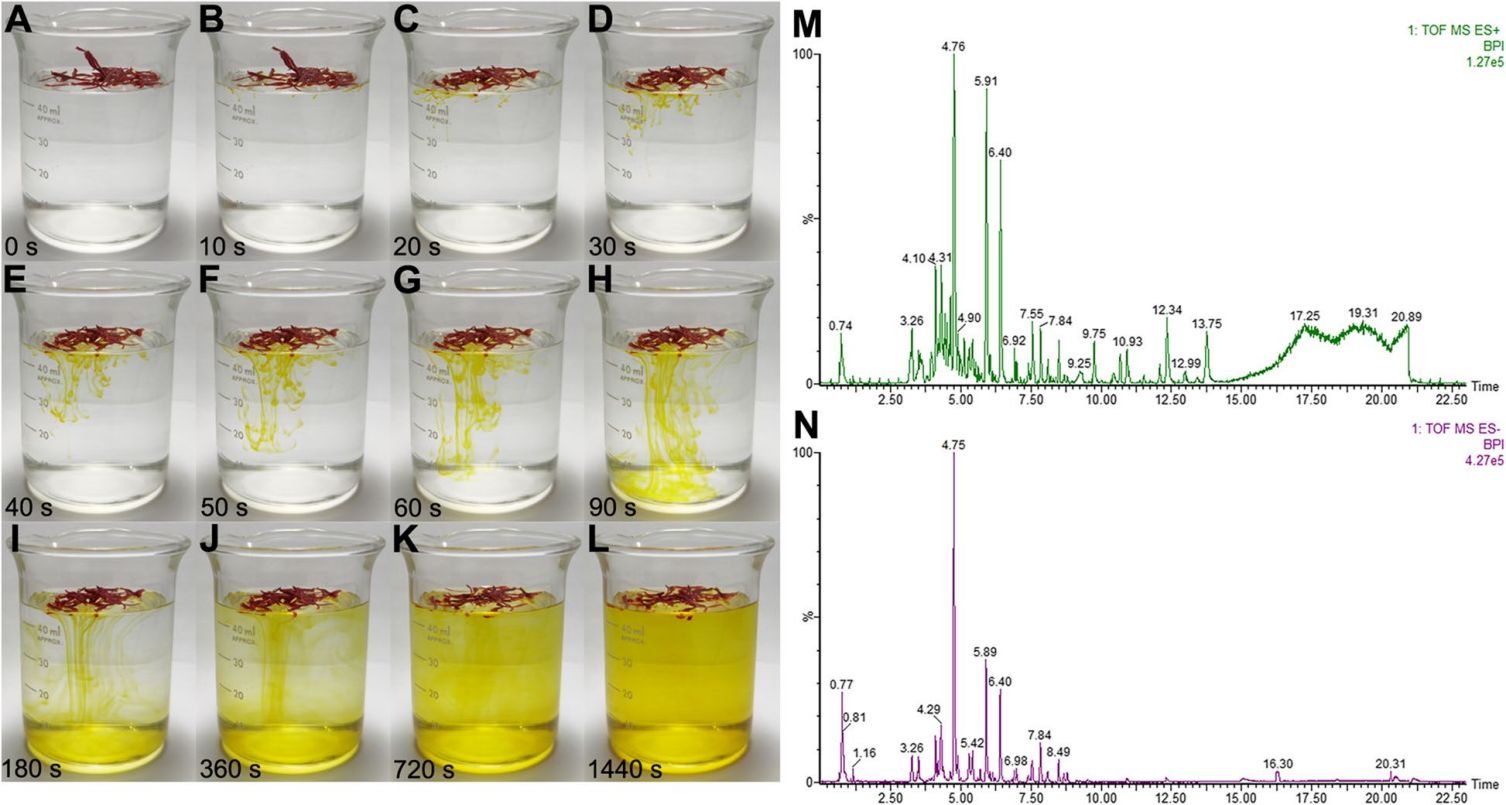
**A** Identification of saffron in water test. Serial still images were recorded at each time point after some saffron was floated on the water surface. **B** Representative total ion chromatogram of HPLC/MS analysis of Ethanol extract of saffron (EES)

The water test of saffron is a traditional method for authenticity identification. After a few saffrons were gently placed on the water surface, then the golden oily liquid was observed floating on the water surface and multiple golden lines reached the bottom of the beaker from the water surface. After 720 s, all the water became a golden yellow clear solution (Fig. 1A-L).

### Identification of neuroprotective composition in EES

Figure 1M and N show that chemical base peak intensity (BPI) chromatogram of key compounds characterization of saffron in positive ion mode and negative ion mode determined by UPLC-Q-TOF/MS. Following the accurate molecular data, 10 chromatographic peaks have been determined using UPLC-QTOF/HDMS (Fig. 1M and N). Identification of compounds was achieved by comparison to mass spectral data in the available literature. The data were presented in Table 1.

**Table 1:** Identification of differential compounds based on UPLC-MS

t_R_ (min)	Identified compound	Mass (neutral)	Error (ppm)	Formulate	MS^E^ fragment ions (m/z)
3.26	Picrocrocinic acid	346.1628	-1.16	C_16_H_26_O_8_	345.1546[M-H]^-^
3.5	Astragalin	448.1006	0.00	C_21_H_20_O_11_	449.1084[M+H]^+^
3.5	Kaempferol-3-O-β-D-sophorocoside	610.1534	-3.11	C_27_H_30_O_16_	611.1593[M+H]^+^
4.12	Rutin	610.1534	7.04	C_27_H_30_O_16_	611.1655[M+H]^+^
4.57	Quercetin-3-O-sophoroside	626.1483	5.44	C_27_H_30_O_17_	625.1439[M-H]^-^
4.76	Safranal	150.1045	5.96	C_10_H_14_O	151.1132[M+H]^+^
4.9	Kaempferol	286.0477	-0.35	C_15_H_10_O_6_	287.0554[M+H]^+^
5.89	Crocin I	976.3788	-0.82	C_44_H_64_O_24_	975.3702[M-H]^-^
6.38	Crocetin	328.1675	-0.61	C_20_H_24_O_4_	329.1751[M+H]^+^, 311.1652[M+ H-H_2_O]^+^
8.1	Crocin II	814.3259	-2.58	C_38_H54O_19_	815.3160[M-H]^-^

### Effect of EES on PC12 cell viability

**Fig. 2:**
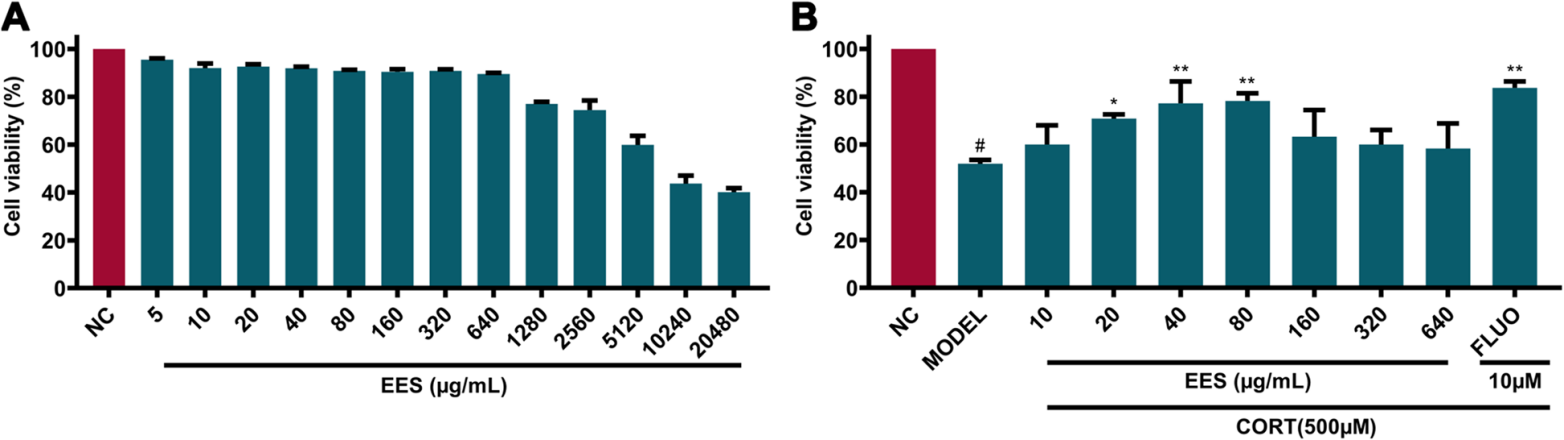
Cytotoxicity of EES on PC12 cell viability (**A**) and protective efficacy (**B**) from corticosterone treated PC12 cell injury. Data are presented as mean ± SD (*n* = 3). ^#^ Indicates *p <* 0.05 as compared with NC group, * indicates *p <* 0.05 or ** indicates *p <* 0.01 as compared with Model group

Compared with the negative control group, different concentrations of EES (5, 10, 20, 40, 80, 160, 320, 640 μg/mL) showed low cytotoxicity to PC12 cells, with cell viability of 95.56 ± 0.54, 92.01 ± 1.96, 92.66 ± 0.97, 91.97 ± 0.65, 90.85 ± 0.48, 90.47 ± 1.10, 90.83 ± 0.69, and 89.53% ± 0.48%, respectively, without statistical difference among these groups. The toxicity of 1280, 2560, 5120, 10,240, and 20,480 μg/mL EES to PC12 cell began to increase by progressively, and the cell viability were 77.06 ± 0.83%, 74.46 ± 4.00%, 59.87 ± 3.84%, 43.70 ± 3.37%, and 40.15 ± 1.70% (Fig. 2A), respectively. The results showed that concentrations below 640 μg/mL are safe doses of EES.

### Protective effect of ESS in corticosterone-induced PC12 cell

As shown in Fig. 2B, treatment with 500 μM of corticosterone for 24 h had been shown to cause cytotoxicity in PC12 cells. Nevertheless, different concentrations of EES (20, 40, and 80 μg/mL), significantly increased the cell viability by 70.83 ± 1.77% (*P* < 0.05), 77.28 ± 9.11% (*P* < 0.01), and 78.24 ± 3.17% (*P* < 0.001), respectively. As a positive control, 10 μM FLUO revealed a significant increase the cell viability by 83.702 ± 2.71% (*P* < 0.001) under 500 μM corticosterone co-incubation.

### EES alters RNA expression profiles in PC12 cell

**Fig. 3:**
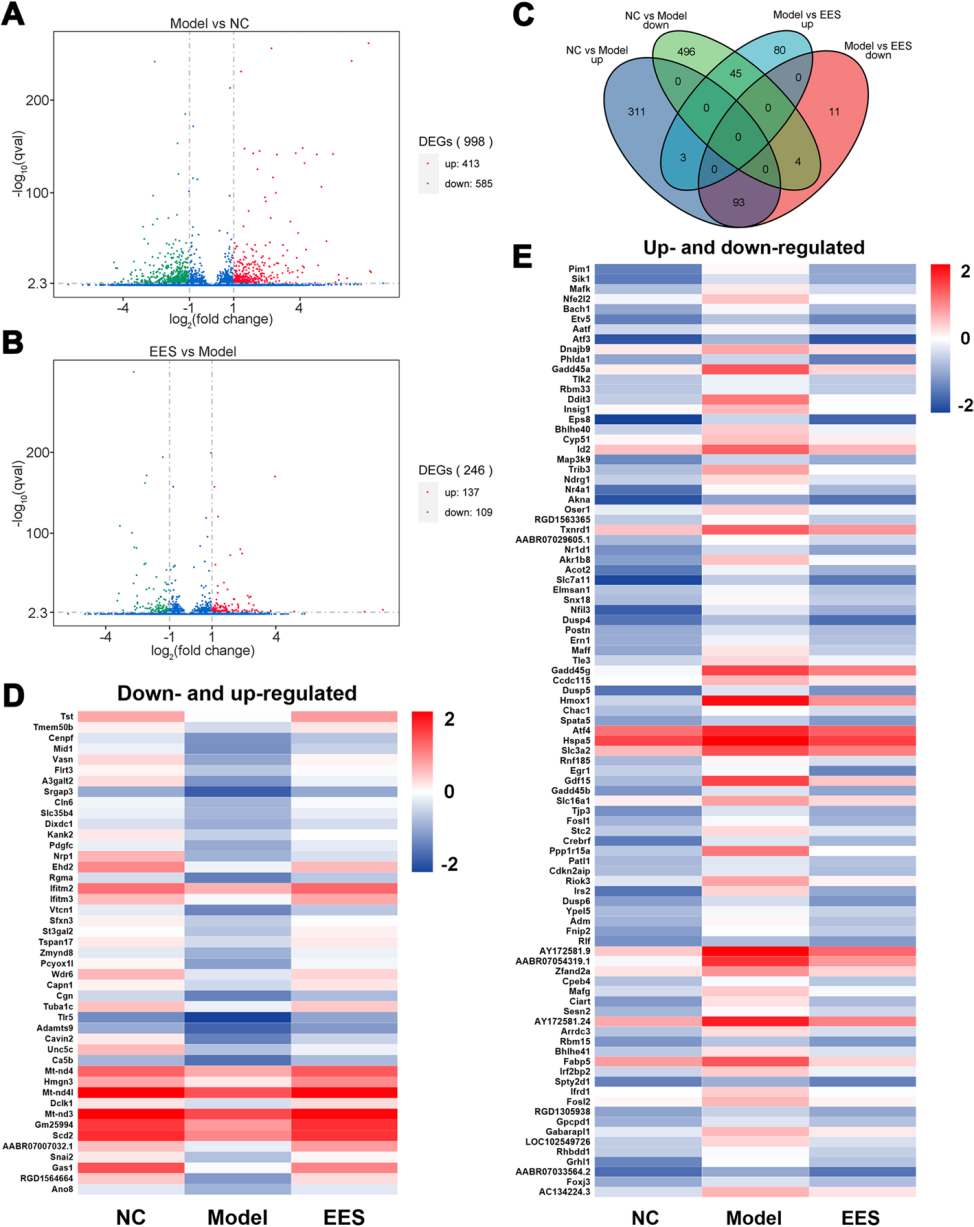
Differential expression of RNAs in CORT-induced PC12 cells by EES treatment. Volcano plots (**A** and **B**) were used to visualize the differential expression between NC versus Model groups, and EES versus Model groups severally. X-axis: Log2-fold change of RNA expression levels. Y-axis: q-value (−log10 transformed, adjusted *p*-value) of the RNA. The Venn diagram (**C**) summarizes comparably and differentially expressed genes, whereas the heat map (**D** and **E**) provides a global view of differentially expressed genes. Colors correspond to significant fold change expression. Red, high expression; blue, low expression

To further investigate the molecular mechanisms of EES, RNA expression profiles in PC12 cells were analyzed by high-throughput RNA sequencing technology as introduced above. As shown in Fig. 3A and B, compared with the NC group, there were 998 DEGs in the Model group, including 413 up-regulated and 585 down-regulated DEGs. Compared with the Model group, there are 246 DEGs in the EES group, including 137 up-regulated and 109 down-regulated DEGs. One hundred thirty-eight DEGs were further detailed by plotting DEGs Venn diagrams (Fig. 3C) and heatmap (Fig. 3D and E). Forty-five DEGs were down-regulated after modeling and up-regulated after EES intervention. Reversely, 93 DEGs were up-regulated after modeling and down-regulated after EES administration.

### GO enrichment analysis of the DEGs

**Fig. 4:**
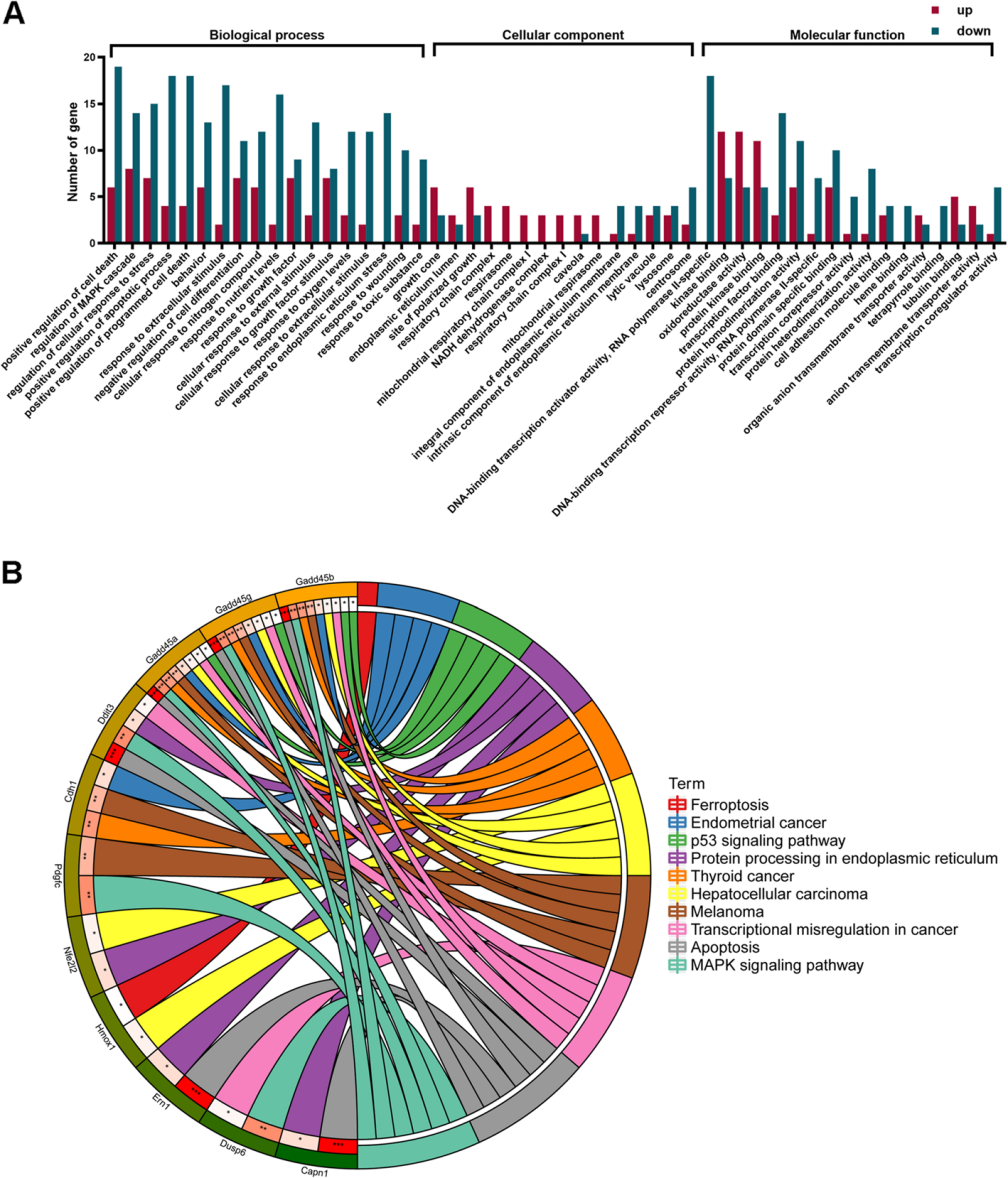
**A** GO terms of DEGs. The results are summarized in three main categories: biological process, cellular component, and molecular function. The X-axis indicates the second level term of gene ontology; The Y-axis shows the number of genes. Up-regulated DEGs were represented by red, and down-regulated DEGs were remarked with the green column. **B** 10 enriched pathways annotated in the KEGG database of DEGs. The outermost circle is the term on the right, the color corresponding to the gene on the left is the gene’s expression multiple, and the inner circle on the left represents the significant *p*-value of the corresponding pathway of the gene

GO functional enrichment analysis was performed for the DEGs identified by RNA-seq. and the 138 DEGs were divided into three groups according to their functions and biological pathways, including the biological processes, cellular components, and molecular functions. The results were shown in Fig. 4A.

### KEGG pathway analysis of DEGs

The 138 DEGs were retrieved by the KEGG database, and the results showed that the DEGs were mainly assigned in the 10 pathways (Fig. 4B, p<0.05). KEGG pathway analysis helps provide an in-depth understanding of the biological mechanisms of genes. KEGG analysis showed that two most of the DEGs were assigned in mitogen-activated protein kinase (MAPK) signaling.

### Validation of the RNA-seq results by qPCR

**Fig. 5:**
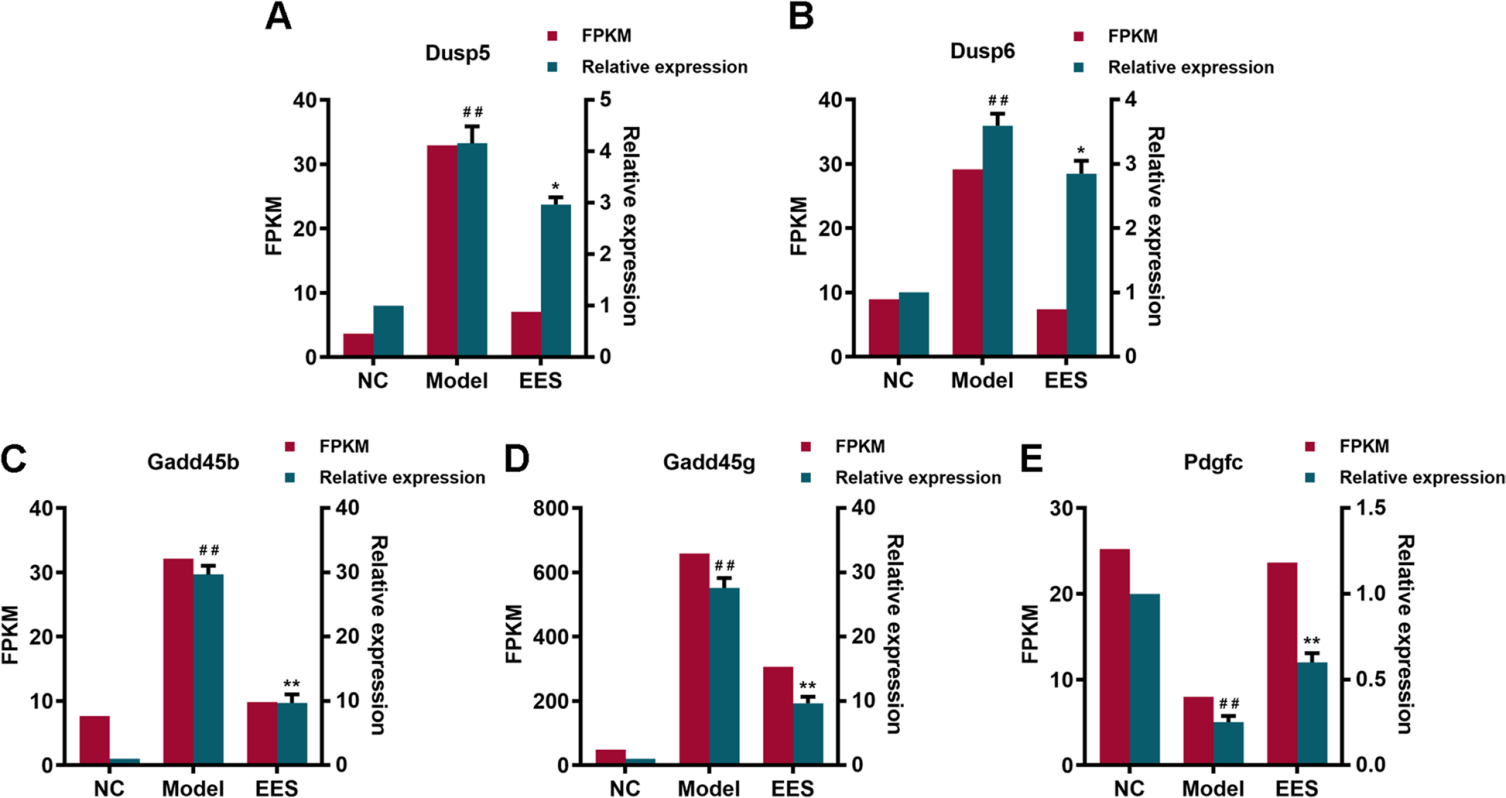
Validation of RNA-seq results with RT-qPCR. Confirmation of expression patterns for 5 genes involved in the Dusp5, Dusp6, Gadd45b, Gadd45g, and Pdgfc using qPCR. FPKM values from the RNA-seq are shown in the red column, qPCR values are shown in the green column. Data are presented as mean ± SD (*n* = 3). ^##^ Indicates *p <* 0.01 as compared with NC group, * indicates *p <* 0.05 or ** indicates *p <* 0.01 as compared with Model group

To verify the accuracy and reproducibility of RNA-seq results, five highly expressed DEGs were selected to confirm their expression levels by qPCR (Fig. 5A-E). The trend of expression changes of these selected genes based on qPCR was similar to those detected by the RNA-seq method, which corroborated the reliability and validity of the RNA-seq technology. The primers’ design was shown in Table 2.

**Table 2:** Primer sequences used for qPCR analysis

Gene name	Forward (5′- 3′)	Reverse (5′- 3′)
Dusp5	CACACCGCCGACATTAG	GCTCCTCCTCTGCTTGA
Dusp6	TGCCAAGGACTCTACTAACT	CACCAGGACACCACAGT
Gadd45b	GAAGAAGAGGAGGATGATATTG	CAGTTCGTGACCAGGAG
Gadd45g	GTTGATCCAGGCGTTCT	GGTCCTTCCATGTGTCTT
Pdgfc	GGCAGATAGACTTGGACAG	CCGTATGGACACAGAGAAG
GAPDH	GTTCAACGGCACAGTCA	CTCAGCACCAGCATCAC

## Discussion

Depression is one of the most common forms of mental illness and the leading cause of disability in a variety of conditions [18]. Approximately 350 million people worldwide suffer from the anxiety and pain caused by depression [19]. Depression affects the quality of life of patients, caregivers, and family members, and thus increases the cost of public health care annually [20]. However, little is known about the pathogenesis of depression. One of the main reasons is the limited availability of validated depression models. These experiments are related to ethical issues and are costly, laborious, and often produce ambiguous results [21]. In contrast, in vitro assay methods are relatively rapid and less demanding methods for testing the neurotoxicity profiles of chemicals. In vitro techniques for studying neurodegenerative disorders are based primarily on chemically induced rodent or human cell lines, which in turn can reveal fundamental, specific changes to individual cells or related molecular pathways [22].

It is estimated that one in 20 people worldwide suffers from depression [23], and although prevalence varies by region and country, depression is common worldwide. However, timely diagnosis and treatment of patients with depression can play an important role in reducing disability, improving the survival and quality of life of patients, and helping to save financial resources in national health sectors [24]. Classical antidepressants involve the regulation of the serotonergic and norepinephrine systems. However, these antidepressants are unsatisfactory because of their slow onset and side effects that sometimes reduce patient compliance, limiting their efficacy [25]. In contrast, many commonly used traditional Chinese medicinal herbs for clinical treatment are safer and have fewer side effects, and can be considered an effective alternative treatment for depression [26].

Many significant pharmacological effects of saffron have been recognized by current research, and one of the most confirmed findings on the benefit of saffron is its antidepressant activity. Clinical trials have been conducted to evaluate the efficacy of saffron in the treatment of mild to moderate depression. The study reported that saffron was more effective than placebo, at least equivalent to the therapeutic doses of imipramine and fluoxetine [27]. Saffron, a perennial herb of the Iridaceae family, is originally considered a spice. However, as a drug, the chemistry of saffron is complicated; this spice has primary metabolites, which are a large number of compounds belonging to different classes of secondary metabolites, products of metabolism not ubiquitous but essential for the development or reproduction of the organism, such as carotenoids, monoterpenes, and flavonoids, including main anthocyanins in nature, such as carbohydrates, minerals, fats, vitamins, amino acids, and proteins [28].

The exact mechanism of saffron in the treatment of depression is still unclear. We performed high-throughput screening for its potency using transcriptomics and validated the transcription levels of several signature differential genes. Through KEGG pathway enrichment analysis, we found that the DEGs were chiefly assigned in the pathway of MAPK signaling pathway. MAPK superfamily is a cell-mediated signaling pathway, which connects inputs to cells, and changes in gene expression led to the modifications in cell phenotype. To mediate its function, the MAPK signaling pathway consists of three kinases, each of which consists of multiple members, namely, extracellular regulated kinases (ERKs), Jun amino-terminal kinase/stress-activated kinase (JNKs/ SAPKs), and p38 kinase. The MAPK signaling pathway is involved in such processes as gene transcription, mRNA translation, protein stability, protein localization, and enzyme activity, thus regulating cell proliferation, cell differentiation, cell survival, and cell death [29]. Markedly, the MAPK pathway has been shown to play a pivotal role in depression and antidepressants [29, 30].

.The largest histone phosphatase that specifically regulates MAPK activity in mammalian cells is the Dual-specificity phosphatases (DUSPs) family phosphatase [31]. DUSPs constitute a large heterogeneous subgroup of the type I cysteine-based protein-tyrosine phosphatase superfamily. DUSPs are characterized by their ability to dephosphorylate both tyrosine and serine/threonine residues. The protein encoded by this gene is a member of the dual-specificity protein phosphatase subfamily. These phosphatases inactivate their target kinases by dephosphorylating both the phosphoserine/threonine and phosphotyrosine residues. They negatively regulate members of the MAPK superfamily (MAPK/ERK, SAPK/JNK, p38), which are associated with cellular proliferation and differentiation. Different members of the family of dual specificity phosphatases show distinct substrate specificities for various MAP kinases, different tissue distribution and subcellular localization, and different modes of inducibility of their expression by extracellular stimuli [32]. Respectively, DUSP5 and DUSP6 inactivate ERK1 and ERK2. DUSP5 is expressed in a variety of tissues with the highest levels in the pancreas and brain and is localized in the nucleus. DUSP6 is expressed in a variety of tissues with the highest levels in the heart and pancreas, and unlike most other members of this family, is localized in the cytoplasm. Mutations in this gene have been associated with congenital hypogonadotropic hypogonadism. Alternatively, spliced transcript variants have been found for this gene [33].

.The growth arrest and DNA damage-inducible protein GADD45 (Gadd45) protein is involved in several cellular mechanisms, including cell cycle control, DNA damage sensation and repair, genotoxic stress, oncology, and molecular epigenetics. These include early and postnatal development, injury, cancer, memory, aging, neurodegenerative diseases, and psychiatric conditions. These proteins act through a variety of molecular signal cascades and are important constituent members of the MAPK signaling pathway [34]. Both the GADD45B gene and GADD45G gene is member of a group of genes whose transcript levels are increased following stressful growth arrest conditions and treatment with DNA-damaging agents. The genes in this group respond to environmental stresses by mediating activation of the p38/JNK pathway. This activation is mediated between their proteins binding and activating MTK1/MEKK4 kinase, an upstream activator of both p38 and JNK MAPKs. The function of these genes or their protein products is involved in the regulation of growth and apoptosis. These genes are regulated by different mechanisms, but they are often coordinated expressed and can function cooperatively in inhibiting cell growth [35]. Ma et al. [36] identified GADD45B as a neural activity-induced immediate early gene in mature hippocampal neurons. Mice with Gadd45b deletion exhibited specific deficits in a neural activity-induced proliferation of neural progenitors and dendritic growth of newborn neurons in the adult hippocampus. Mechanistically, GADD45B is required for activity-induced DNA demethylation of specific promoters and expression of corresponding genes critical for adult neurogenesis, including brain-derived neurotrophic factor (BDNF) and fibroblast growth factor (FGF). Thus, Ma et al. concluded that GADD45B links neuronal circuit activity to epigenetic DNA modification and expression of secreted factors in mature neurons for extrinsic modulation of neurogenesis in the adult brain. Zhang et al. [37] found that the mRNA expression of the GADD45G gene is significantly different between normal pituitary tissue and clinically nonfunctioning pituitary adenomas using cDNA-representational difference analysis. The authors concluded that GADD45G is a powerful growth suppressor controlling pituitary cell proliferation and that it represents the first identified gene whose expression is lost in the majority of pituitary tumors.

Platelet-derived growth factor (PDGF)-induced DNA synthesis and proliferation involves activation of Ras and MAPK. Cross-talk between protein kinase A (PKA) signaling and tyrosine-kinase receptor signaling results in PKA inhibition of the MAP kinase cascade, probably at the level of Raf [38]. The protein encoded by this gene is a member of the platelet-derived growth factor family. The four members of this family are mitogenic factors for cells of mesenchymal origin and are characterized by a core motif of eight cysteines. This gene product appears to form only homodimers. It differs from the platelet-derived growth factor-alpha and beta polypeptides in having an unusual N-terminal domain, the CUB (complement C1r/C1s, Uegf, Bmp1) domain. Alternatively, spliced transcript variants have been found for this gene [39]. Fredriksson et al. [39] found that tissue plasminogen activator (tPA), a serine protease, specifically cleaved and activated latent PDGFCC by interacting with the PDGF/ vascular endothelial growth factor (VEGF)-like growth factor domain. The growth of primary fibroblasts in culture was dependent on tPA-mediated cleavage of latent PDGFCC, which generated a growth stimulatory loop. Su et al. [40] demonstrated that intraventricular administration of tPA in mice increased cerebrovascular permeability via activation of Pdgfcc and its receptor Pdgfra. Morphologic changes were observed primarily in arterioles. Immunohistochemical studies showed that Pdgfcc was localized to arterioles in the cortex, striatum, and hippocampus, as well as additional brain regions, and closely resembled tissue distribution of tPA. The Pdgfra receptor was located primarily on perivascular astrocytes associated with arterioles. In a mouse model of ischemic stroke with tPA administration, treatment with the PDGFR-alpha inhibitor imatinib resulted in decreased cerebrovascular permeability and reduced lesion volume. Su et al. suggested that the known association of hemorrhagic complications in some patients with ischemic stroke treated with tPA may be due in part to the activation of PDGFCC by therapeutic tPA. The findings also indicated that PDGF signaling regulates blood-brain barrier permeability.

Although we have preliminarily discussed the protective mechanism of EES on PC12 cells, we recognize that there are some limitations in this study. Firstly, our research still stays at the level of in vitro and indirectly constructs the in vitro model of PC12 cell injury, which can only partially suggest the changes of its biological function. Therefore, we still need to further construct an animal model for in-depth exploration. Secondly, the research on the molecular mechanism currently stops at the transcriptional level and does not go deep into the protein level, so it is impossible to explain the exact mechanism of EES. Therefore, to more clearly explain the pharmacodynamic mechanism of EES, more protein-level experimental studies need to be carried out to reveal the effects of EES on protein, protein-protein, and signal pathways. Finally, if we can carry out clinical randomized controlled trials and obtain patient samples to further verify the speculation and results of this experiment, then we can truly provide reference evidence for the clinical treatment of depression.

## Conclusions

In summary, we have found in this study that EES can improve CORT-induced PC12 cell injury, while accompanied by significant changes in RNA expression in PC12 cells. Among them, MAPK pathway genes Dusp5, Dusp6, Gadd45b, Gadd45g, and Pdgfc have undergone noticeable changes. This study helps to understand the potential mechanism of depression, as the damage to neuronal cell survival and neurite growth caused by CORT undoubtedly adversely affects neuronal cell function and signal transduction. Therefore, they may be potential molecular targets of EES in the treatment of depression, providing valuable insights into the treatment or diagnosis of depression.

## Data Availability

The datasets generated and analyzed during the current study are available in the Metascape repository [https://metascape.org].

## Abbreviations

EES: Ethanol extract of saffron
CORT: Corticosterone
SSRIs: Selective serotonin reuptake inhibitors
HPA: Hypothalamic-pituitary-adrenal
CCK-8: Cell Counting Kit-8
DEGs: Differentially expressed genes
UPLC: Ultra Performance Liquid Chromatography
MS: Mass Spectrometry
ESI-Q-TOF-MS: Electrospray ionization Quadrupole Time-of-Flight Mass Spectrometer
GO: Gene Ontology
KEGG: Kyoto Encyclopedias of Genes and Genomes
SD: Standard deviation
MAPK: Mitogen-activated protein kinase
ERKs: Extracellular regulated kinases
JNKs/ SAPKs: Jun amino-terminal kinase/stress-activated kinase
DUSPs: Dual-specificity phosphatases
Gadd45: Growth arrest and DNA damage-inducible protein GADD45
BDNF: Brain-derived neurotrophic factor
FGF: Fibroblast growth factor
PDGF: Platelet-derived growth factor
PKA: Protein kinase A
CUB: Complement C1r/C1s, Uegf, Bmp1
tPA: Tissue plasminogen activator
VEGF: Vascular endothelial growth factor
